# A magnesium transporter is involved in the cesium ion resistance of the high-concentration cesium ion-resistant bacterium *Microbacterium* sp. TS-1

**DOI:** 10.3389/fmicb.2023.1136514

**Published:** 2023-02-23

**Authors:** Yoshiki Ishida, Takahiro Koretsune, Eri Ishiuchi, Miyu Teshima, Masahiro Ito

**Affiliations:** ^1^Graduate School of Life Sciences, Toyo University, Oura-gun, Gunma, Japan; ^2^Faculty of Life Sciences, Toyo University, Oura-gun, Gunma, Japan; ^3^Bio-Nano Electronics Research Center, Toyo University, Kawagoe, Saitama, Japan; ^4^Bio-Resilience Research Project (BRRP), Toyo University, Oura-gun, Gunma, Japan

**Keywords:** cesium-resistant microorganism, magnesium transporter, *Microbacterium*, alkaliphile bacteria, alkaliphiles, jumping spider, Cs^+^/H^+^ antiporter

## Abstract

Cesium ion (Cs^+^) resistance has been reported in bacteria but is poorly understood as reports on Cs^+^-resistant bacteria have been limited. We previously reported a novel Cs^+^/H^+^ antiporter CshA implicated in Cs^+^-resistance in *Microbacterium* sp. TS-1. The present study used the same screening method to isolate novel Cs^+^-sensitive mutants and their revertants from TS-1. A comparative mutation site analysis using whole-genome sequencing revealed that *MTS1_03028* encodes the Mg^2+^ transporter MgtE and is a candidate Cs^+^ resistance-related gene. We performed a bioinformatic analysis of *MTS1_03028* and complementation experiments on Cs^+^ resistance in the TS-1 MTS1_03028 mutants Mut5 and Mut7 as well as *Escherichia coli* expressing *MTS1_03028* in the presence of Mg^2+^. We established the role of MgtE in Cs^+^ resistance through a functional analysis of TS-1. Enhancing Mg^2+^ transport by expression of MTS_03028 conferred increased Cs^+^ resistance. When this strain was exposed to Cs^+^ concentrations exceeding 200 mM, CshA consistently lowered the intracellular Cs^+^ concentration. To our knowledge, the present study is the first to clarify the mechanism of Cs^+^ resistance in certain bacteria. The study findings offer important insights into the mechanism of bacterial resistance to excess Cs^+^ in the environment, suggesting the potential for bioremediation in high Cs-contaminated areas.

## 1. Introduction

Cesium (Cs) has received global attention because large amounts of its radioactive isotopes (^134^Cs and ^137^Cs) were released into the environment after the 1986 Chernobyl and 2011 Fukushima nuclear power plant accidents (Buesseler et al., 2012; Hirose, 2016; Vasylenko et al., 2021; Nakamura et al., 2022; Wu et al., 2022). Since the latter half of the 2010s, there has been an increase in the amount of research on radioactive Cs^+^ contamination of the soil (Sakai et al., 2021; Singh et al., 2022), decontamination and bioremediation efforts (Liu et al., 2014; Singh et al., 2022), and the quest to identify Cs^+^-resistant microorganisms (Dekker et al., 2014; Kato et al., 2016; Swer et al., 2016; Zhang et al., 2021). By utilizing the cesium resistance mechanism of Cs^+^-resistant microorganisms, it can be used by imparting high-concentration cesium-tolerant functions to radioresistant bacteria with low Cs^+^-resistant performance; it is thought that this will lead to the creation of highly functional radioresistant bacteria that efficiently recover radioactive cesium from radioactive cesium-contaminated environments. Therefore, such highly functional microorganisms can be used for bioremediation in contaminated environments.

As the chemical properties of Cs^+^ resemble those of potassium (K^+^), erroneous cellular Cs^+^ influx through the K^+^ uptake system may occur in microorganisms and animal and plant cells and inhibit their growth (Hampton et al., 2004; Kato et al., 2016). Prior studies on Cs^+^ cytotoxicity in *Escherichia coli* reported that Cs^+^ erroneously entered the cells *via* the K^+^ uptake system and intracellularly accumulated as the bacterium lacks a Cs^+^ efflux mechanism. Hence, the intracellular Cs^+^ content increased over time (Bossemeyer et al., 1989). Moreover, K^+^ homeostasis and cellular turgor are maintained by the transport of K^+^ out of the cell *via* the K^+^ excretion system. It has been reported that *E. coli* growth declines with low intracellular K^+^ concentration.

*Microbacterium* sp. TS-1 was isolated from Jumping Spider ground extract in 2012, and its genomic information was reported in 2013 (Fujinami et al., 2013). This bacterium is a facultative alkaliphilic bacterium with a growth pH range of 6.0 to 10.0 and an optimum pH of 8.0 to 9.0. In addition, it can withstand up to 1.2 M CsCl, making it a high-concentration Cs^+^-resistant bacterium (Koretsune et al., 2022). Previously, the upper growth limit of CsCl concentration for Cs^+^-resistant bacteria was 700 mM for *Bacillus* sp. strain C700 (Zhang et al., 2021). Therefore, strain TS-1 was expected to have a unique Cs^+^ resistance mechanism.

Although there are multiple reports of isolation of Cs^+^-resistant bacteria, the mechanism of their Cs^+^ resistance remains unknown (Dekker et al., 2014; Kato et al., 2016; Swer et al., 2016; Ito and Hasunuma, 2022a,b; Yukawa et al., 2022). We reported that a Cs^+^/H^+^ antiporter called CshA is involved in Cs^+^ resistance in *Microbacterium* sp. TS-1 (Koretsune et al., 2022). In addition to this, another Cs^+^ resistance mechanism was expected.

Herein, we aimed to isolate novel Cs^+^-sensitive mutant strains through continuous screening and identify candidate Cs^+^ resistance-related genes *via* spontaneous mutagenesis and next-generation sequencing. The discoveries made herein could elucidate the mechanisms by which bacteria adapt to cesium ion exposure.

## 2. Materials and methods

### 2.1. Bacterial strains and plasmids

The bacterial strains and plasmids used in the present study are listed in Table 1. The primers used in this investigation are available upon request to the corresponding author. Whole-genome sequencing (WGS) was previously performed on the alkaliphilic *Microbacterium* sp. TS-1 strain (Fujinami et al., 2013).

**Table 1:** Bacterial strains and plasmids used in the present study.

Strain	Genotype	References
*Microbacterium* sp. TS-1	Wild type	Fujinami et al. (2013)
Mut3	Cs^+^-sensitive mutant from TS-1, *MTS1_00475* (*cshA*)	Koretsune et al. (2022)
Mut4	Cs^+^-sensitive mutant from TS-1, *MTS1_00475* (*cshA*)	Koretsune et al. (2022)
Mut5	Cs^+^-sensitive mutant from TS-1	This study
Mut5R	Cs^+^-resistant revertant from Mut5	This study
Mut7	Cs^+^-sensitive mutant from TS-1	This study
Mut7R	Cs^+^-resistant revertant from Mut7	This study
*Escherichia coli*		
KNabc	*ΔnhaA*, *ΔnhaB*, *ΔchaA*, Kan^+^, Ery^+^, Cam^+^, *supE*, *hsd*, *Δ*5*thi*, *Δ* (*lac-proAB*)/F′, [*traD36*, *proAB*^+^, *lacLq*, *lacZ*, ΔM15]	Nozaki et al. (1998)
Mach1	F^−^, [φ80*lac*ZΔM15], Δ*lac*X74, *hsd*R, (r_K_^−^, m_K_^+^), Δ*rec*A1398, *end*A1, *ton*A	Thermo Fisher Scientific, Waltham, MA, United States
Plasmid		
pBAD24	Cloning expression vector, P_BAD_ promoter, Ap^R^	Guzman et al. (1995)
pBAD-00475	pBAD24 carrying *MTS1-00475* (*E. coli* codon-optimized sequence)	Koretsune et al. (2022)
pGEM7zf (+)	Cloning vector; Ap^R^	Promega, Madison, WI, United States
pGEM-03028	pGEM7zf (+) carrying *MTS1*_ *03028* (*E. coli* codon-optimized sequence)	This study

### 2.2. Growth media and conditions

*Escherichia coli* was grown at 37°C in Luria-Bertani (LB) medium (BD Difco™, Franklin Lakes, NJ, United States). Alkaliphilic *Microbacterium* sp. TS-1 was grown at 30°C in neutral complex medium (NC medium) and Tris medium (Imazawa et al., 2016). The latter consisted of 3.63 g L^−1^ Tris base, 1.47 g L^−1^ citric acid monohydrate, 0.5 g L^−1^ yeast extract, 9 g L^−1^ glucose, and 1% (w/v) trace elements (Cohen-Bazire et al., 1957). The solvent was deionized water. The pH was adjusted to 8 and 9 with 1 M *N*-methyl-*D*-glucamine. The pH was adjusted to 7 with 5 N H_2_SO_4_. Tris medium was used for the monovalent cation resistance test as cation influx can be underestimated. The NC medium consisted of 15.5 g L^−1^ K_2_HPO_4_, 4.5 g L^−1^ KH_2_PO_4_, 0.05 g L^−1^ MgSO_4_**•**7H_2_O, 0.34 g L^−1^ citric acid, 5 g L^−1^ peptone, 2 g L^−1^ yeast extract, 5 g L^−1^ glucose, and 11.7 g L^−1^ NaCl. The solvent was deionized water. The final pH was adjusted to the desired value as required with KOH or H_2_SO_4_ (Fujinami et al., 2011). The *E. coli* KNabc transformants were grown in LBK medium (10 g L^−1^ tryptone, 5 g L^−1^ yeast extract, and 6 g L^−1^ KCl; pH 7.5). For growth selection, the medium was supplemented with kanamycin (25 μg mL^−1^) or ampicillin (100 μg mL^−1^). The cells were grown with shaking at 200 rpm and 30°C, and their growth was monitored by measuring OD_600_ in a spectrophotometer UV-1800 (Shimadzu Corporation, Kyoto Japan).

### 2.3. Isolation of novel Cs^+^-sensitive mutants *via* chemical mutation and replica plating

Cs^+^-sensitive strains and their Cs^+^-resistant revertants were generated *via* chemical mutagenesis with ethyl methane sulfonate (EMA), as previously reported (Koretsune et al., 2022). The Cs^+^-sensitive mutants obtained here were designated Mut5 and Mut7. Single colony isolation was performed for the isolated Mut5 and Mut7 on NC agar medium (pH 8). The colonies were inoculated into 2 ml NC medium (pH 8.0) and reciprocally shake-cultured at 200 rpm at 30°C for 18 h. The culture (100 μl) was independently plated on NC agar medium (pH 8) containing 200 mM or 400 mM CsCl to obtain spontaneous mutants whose cesium resistance was restored. Their Cs^+^-resistant revertants obtained here were designated Mut5R and Mut7R.

### 2.4. Cs^+^ resistance test of Cs^+^-sensitive mutants and Cs^+^-resistant revertants

Each TS-1 mutant was isolated from a single colony on NC agar (pH 8.0). Each colony was inoculated into a 14-mL culture tube containing 2 ml neutral composite medium (pH 8.0). The tubes were shaken at 200 rpm and 30°C for 18 h. The culture broth was used as the preculture medium. Two milliliters test medium and 10 μl preculture were placed in each 14-mL culture tube. The tubes were shaken at 200 rpm and 30°C for 16 h. OD_600_ was measured and Cs^+^ resistance was assessed for each strain. Three independent experiments were performed.

### 2.5. Investigation of effects of Mg^2+^ on Cs^+^ resistance in Mut5 and Mut7

Tris medium (pH 8.0) was supplemented with 100–1,200 mM Cs^+^ and it was determined whether Mg^2+^ addition to semisynthetic medium improved Cs^+^ resistance in Mut5 and Mut7. Single colonies were isolated from the TS-1, Mut5, and Mut7 strains on NC agar (pH 8.0). Each TS-1 mutant colony was then inoculated into a culture tube containing 2 ml NC medium and shaken at 200 rpm and 30°C for 18 h. The culture broth was used as the preculture medium. Two milliliters Tris medium was supplemented with various concentrations of Tris medium plus 100–1,200 mM CsCl with or without 2 μl of 1 M MgCl_2_. Then, 10 μl preculture (0.5% (v/v)) was inoculated into the duplicated medium and shaken at 200 ppm and 30°C for 18 h. Then, OD_600_ was measured using a spectrophotometer. Three independent experiments were performed.

### 2.6. TS-1 mutant chromosomal DNA preparation

Single Mut5, Mut5R, Mut7, and Mut7R colonies were each inoculated into 2 ml NC medium (pH 8.0) and shaken at 200 rpm and 30°C for 18 h. Five hundred microliters preculture was inoculated into a 24φ test tube containing 4.5 ml NC medium (pH 8.0) and shaken at 200 rpm and 30°C for 4 h. The entire culture medium was then centrifuged at 9,100 × *g* and 4°C for 5 min and the supernatant was removed. Chromosomal DNA was extracted using a DNeasy Blood and Tissue kit (QIAGEN, Tokyo, Japan) according to the manufacturer’s instructions.

### 2.7. Comparative analysis of WGS data

The chromosomal DNA was subjected to WGS with HiSeq X 2 × 150 bp (Illumina, San Diego, CA, United States) by Eurofins Genomics K.K. (Tokyo, Japan). The genome sequences of the mutant strain were then subjected to single-nucleotide polymorphism (SNP) analysis and the mutation sites were extracted. Variant calls were analyzed using samtools v. 1.6,^1^ and bases differing from the reference were extracted from the mapping results. Variants were filtered using vcfutils.pl. in bcftools v. 1.6^2^ and selected if they met the default settings for the called variants. Genes with different mutation sites were selected as candidates for Cs^+^ resistance. The selected Cs^+^ resistance-related candidate gene was identified from the annotation results for the TS-1 genome sequence. It was then determined whether the mutation caused nonsynonymous amino acid substitution. Mutations were detected in the genes overlapping Cs^+^-sensitive mutant strains. Those with reverse mutations common to both revertant strains were selected as candidate Cs^+^ resistance-related genes. The nucleotide sequence data are available in the DDBJ Sequenced Read Archive^3^ under accession Nos. DRR328007 (Mut5), DRR328008 (Mut5R), DRR328009 (Mut7), and DRR328010 (Mut7R).

### 2.8. Artificial gene synthesis

*MTS1_03028* was optimized for *E. coli* codons with GENEius,^4^ and each identified gene was artificially synthesized by Eurofins Genomics. The gene sequence was registered in the DNA Data Bank of Japan (DDBJ).^5^ The accession number for *MTS1_03028* is LC655172.

### 2.9. Alignment of the Mg^2+^ transporter-related genes with protein homologs in several bacterial species

The amino acid sequences of the Cs^+^ resistance-related candidate genes and their homologs were obtained using the BLASTP algorithm at NCBI^6^. The amino acid residues selected in the alignment were analyzed using ClustalW^7^ (Larkin et al., 2007). ETE3^8^ was used to construct a phylogenetic tree for MTS1_03028 and its homologs using the neighbor-joining (NJ) method (Huerta-Cepas et al., 2016). Each protein structure was inferred from the amino acid sequence of the Cs^+^ resistance-related genes using TMHMM 2.0.^9^

### 2.10. Construction of plasmids to express *MTS1_03028* optimized for *Escherichia coli* codons

Both 5′ ends of the phosphorylated synthetic *MTS1_03028* fragments optimized for *E. coli* codons were ligated with *Sma*I and pGEM7zf (+) and digested with T4 DNA ligase. The product was then transformed into Mach1-competent *E. coli*. A hundred microliters were spread onto LB agar containing 100 μg/ml ampicillin and statically incubated at 37°C overnight. The plasmids were then isolated and designated pGEM-03028.

### 2.11. Cs^+^ growth test and intracellular Mg^2+^ concentrations for *Escherichia coli* Mach1 transformants subjected to various CsCl concentrations

Single Mach1/pGEM7zf (+) and Mach1/pGEM-03028 colonies were inoculated into 2 ml LB medium (pH 7.5) containing 100 μg/ml ampicillin and shaken at 200 rpm and 37°C for 16 h. Two milliliters LB medium and 50 mM, 100 mM, or 150 mM CsCl plus 100 μg/ml ampicillin were combined with 10 μl preculture. The cultures were shaken at 200 rpm and 37°C for 16 h. OD_600_ was measured in a spectrophotometer and the Cs^+^ resistance of each strain was evaluated. Three independent experiments were performed.

The cultures were then centrifuged at 9,100 × *g* and 4°C for 5 min. The supernatants were removed, and the cell pellets were resuspended in 2 ml of 300 mM sucrose and centrifuged at 9,100 × *g* and 4°C for 5 min. The supernatants were removed, and the cell pellets were suspended in 2 ml of 300 mM sucrose. The cell count was adjusted based on the measured OD_600_. Three microliters of 6 N HCl was added to the suspensions, and they were centrifuged at 9,100 × *g* and 4°C for 5 min. Then, 250 μl Mg assay solution was decanted into a 1.5-mL tube and subjected to the quantitative determination of Mg using a Metallo Assay Magnesium Measurement LS kit (Metallogenics Co. Ltd., Chiba, Japan; Osman et al., 1983) following the manufacturer’s instructions. Then, 3 μl supernatant was added. The reaction was allowed to proceed at 20–25°C for 5 min and OD_600_ was measured. The latter values were used to calculate the intracellular Mg^2+^ concentrations according to the formula and instructions provided by the kit manufacturer.

## 3. Results

### 3.1. Cs^+^-sensitive mutant isolation *via* chemical mutagenesis and replica plating

Mutagenesis was chemically induced by subjecting the TS-1 to 3% (v/v) EMS for 2 h. The cells were cultured until the early stationary phase, spread on NC medium plates, and incubated at 37°C for 2 day. Approximately 41,500 colonies were subjected to replica plating. Two candidate mutants designated Mut5 and Mut7 were sensitive to 200 mM Cs^+^ (Supplementary Figure S1). They were subsequently isolated.

### 3.2. Isolation of Cs^+^-resistant revertants and CsCl growth tests

The reversion mutation rates for Mut5 and Mut7 were 5.8 × 10^−9^ and 1.9 × 10^−8^, respectively.

**Figure 1:**
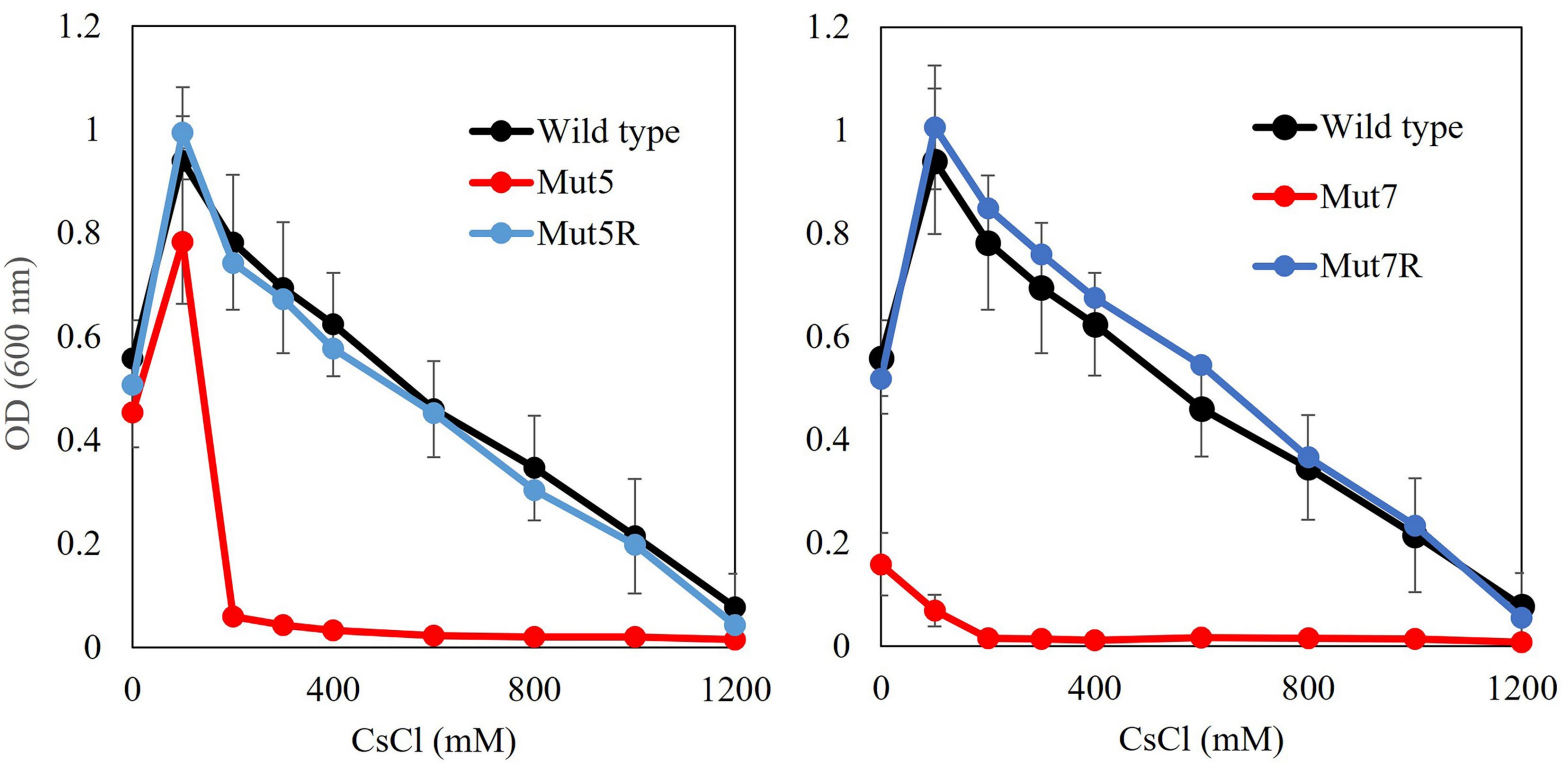
Cs^+^ resistance growth tests on Cs^+^-sensitive mutants and Cs^+^-resistant revertants. Single colonies of each mutant were inoculated into 2 ml NC medium (pH 8.0) and shaken at 200 rpm and 30°C for 18 h. Ten microliters preculture was inoculated into 2 ml Tris medium (pH 8.0) containing 100–1,200 mM CsCl and shaken at 200 rpm and 30°C for 18 h. OD_600_ was measured using spectrophotometry. Error bars show SD for three independent experiments.

CsCl growth tests were performed using the mutant strains (Figure 1). All Cs^+^-sensitive mutants presented with slower growth than the wild type. The revertants recovered the same level of Cs^+^ resistance as the wild type (Table 2).

**Table 2:** Rates of mutation reversion of each Cs^+^-sensitive mutant strain and proteins encoded by genes with revertant mutations.

Mutant	Frequency of Cs^+^-resistant revertant strains	Amino acid mutation site (Cs^+^ sensitive mutant → revertant mutant)	Accession no.
Mut5R	5.8 × 10^−9^	MTS1_00841 phenylalanyl-tRNA synthetase beta subunit (A730V → V730A) (true reversion)	BASQ01000001.1889132–891,651 (minus strand)
		MTS1_02354 putative DNA segregation ATPase (P142S → S142P) (true reversion)	BASQ01000001.12498595–2,499,221
		MTS1_03028 magnesium transporter (T396I → I396T) (true reversion)	BASQ01000002.124174–25,532 (minus strand)
Mut7R	1.9 × 10^−8^	MTS1_03028 magnesium transporter (E310K → K310E) (true reversion)	BASQ01000002.124174–25,532 (minus strand)

Table summarizes the proteins encoded by the genes confirmed to be mutated in the Cs^+^-sensitive mutants and reverted in the Cs^+^-resistant revertants. For genes with mutations and nonsynonymous amino acid substitutions, the mutations are shown in parentheses in the following order: wild type, Cs^+^-sensitive mutant, and Cs^+^-resistant revertant.

### 3.3. Comparative WGS analysis using next-generation sequencing

WGS analyzes indicated that the Cs^+^-sensitive Mut5 and Mut7 harbored 177 and 166 mutations, respectively. They shared a common mutated gene encoding a magnesium transporter designated *MTS1_03028*. Comparison of the genome sequences of Mut5 and the revertant Mut5R confirmed that the latter had the reversion mutation Mut5: T396I → Mut5R: I396T on *MTS1_030285*. In contrast, comparison of the genome sequences of Mut7 and the revertant Mut7R confirmed that the latter had a reversion mutation Mut7: E310K → Mut7R: K310E on *MTS1_030285*. The *MTS1_030285* nucleotides were sequenced for both revertants.

### 3.4. Bioinformatics analysis of MTS1_03028

**Figure 2:**
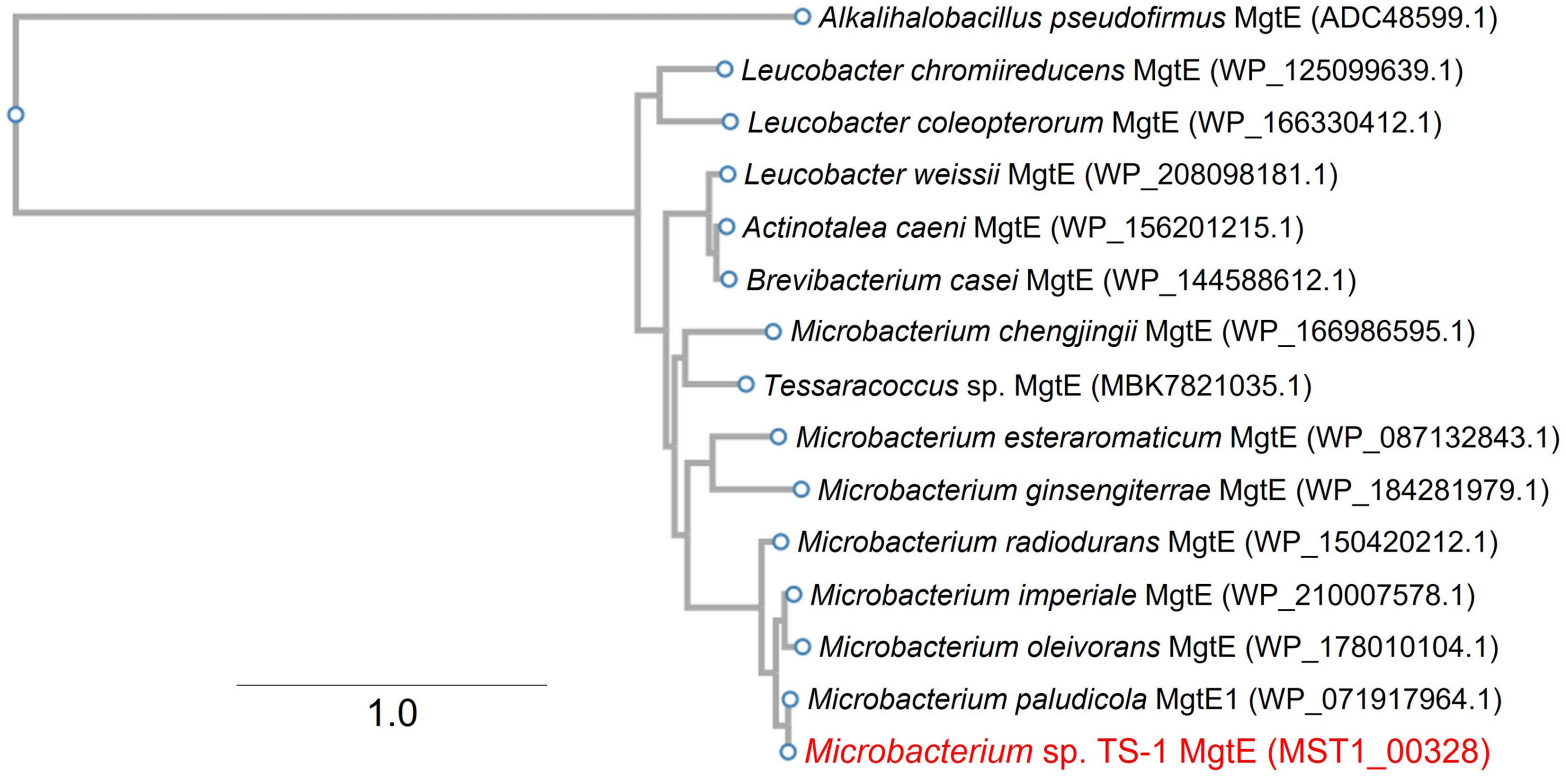
Phylogenetic clustering of MTS1_00328. A phylogenetic tree was constructed based on multiple sequence alignment with MTS1_00328 homolog. Details of this procedure are described in Materials and Methods. MTS1_00328 from *Microbacterium* sp. TS-1 is shown in red. MgtE from gram-positive alkaliphile *Alkalihalobacillus pseudofirmus* was used as an outgroup. The number between branches indicates the bootstrap value. GenBank accession Nos. are provided in parentheses.

**Figure 3:**
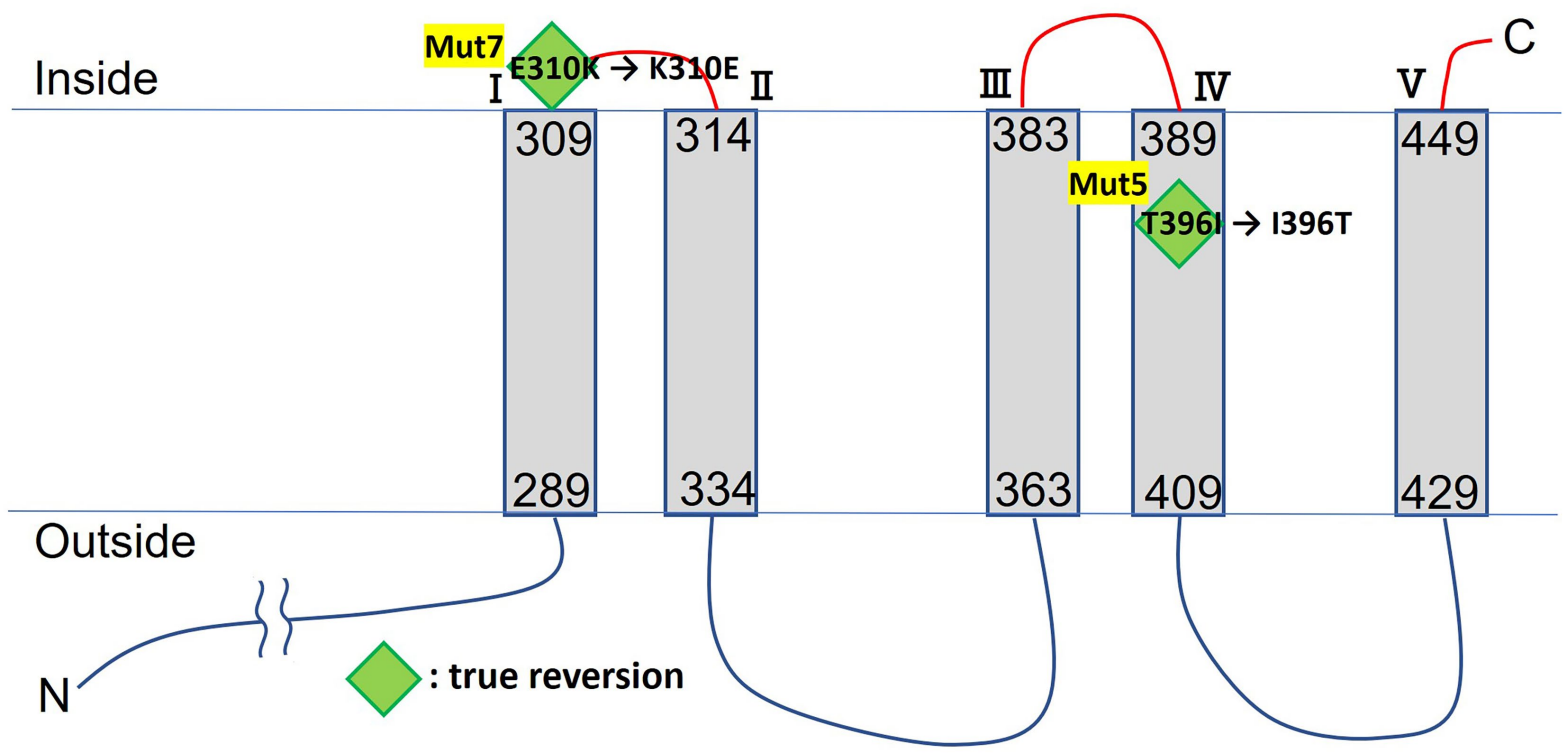
TMHMM transmembrane model of MTS1_00328. Mutation sites of Cs^+^-sensitive mutants are indicated by diamonds. Green diamonds indicate true reversion in Cs^+^-resistant revertant.

Multiple sequence alignment of MTS1_03028 and its protein homologs revealed that it is the Mg^2+^ transporter MgtE (Smith and Maguire, 1998), which is widely distributed among *Microbacterium* spp. (Figure 2). MTS1_03028 showed 99% homology with the MgtE of *Microbacterium paludicola*. Its homologs were detected in *Brevibacterium casei*, *Leucobacter weissii*, and *Actinotalea caeni*, and their amino acid identities were 70, 70, and 69%, respectively. According to protein structure prediction using TMHMM-2.0^10^, MTS1_03028 is a five-transmembrane protein. A schematic diagram of the secondary structure prediction is shown in Figure 3.

### 3.5. Effects of Mg^2+^ on Cs^+^ resistance in the Cs^+^-sensitive TS-1 mutants

**Figure 4:**
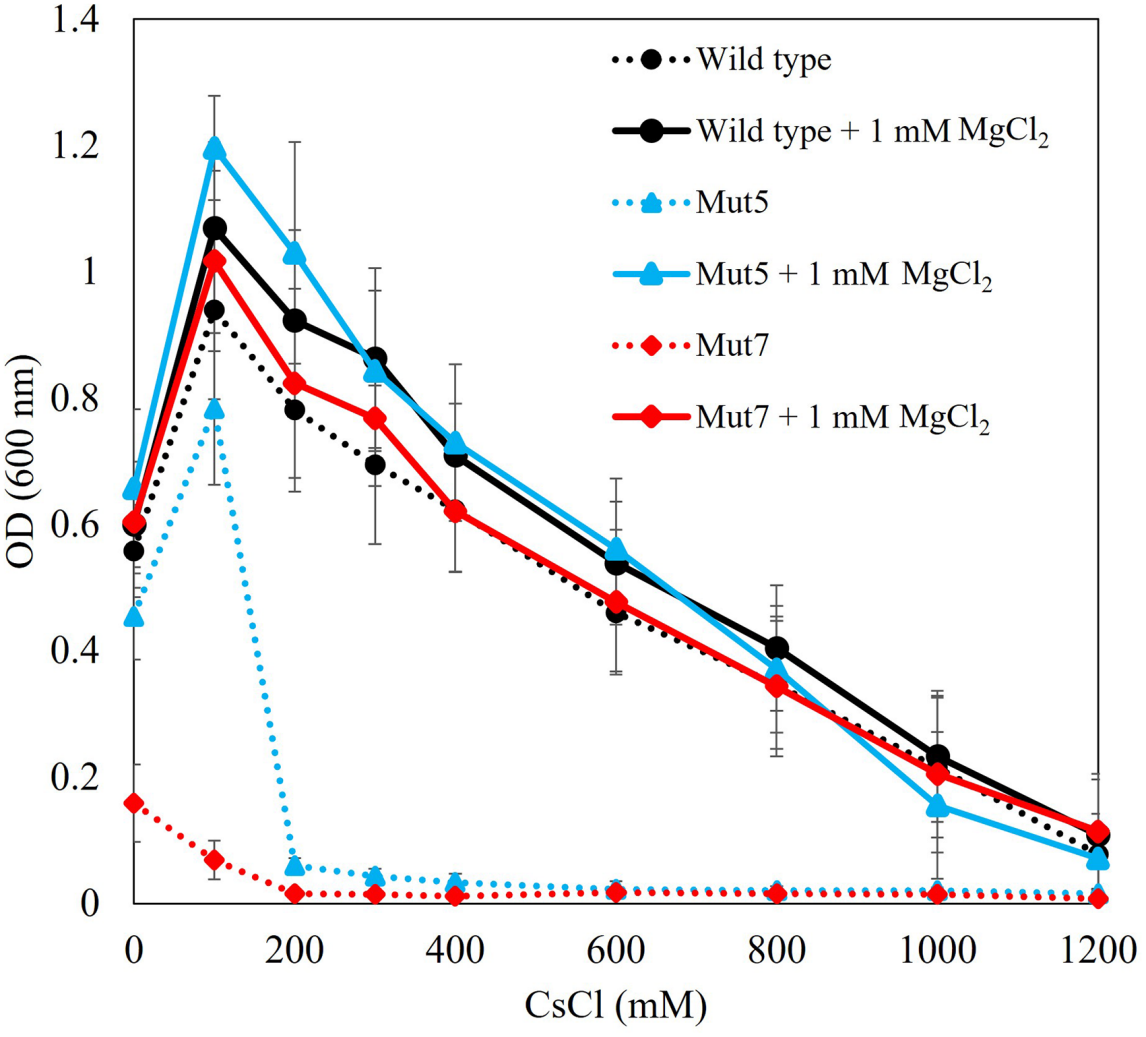
Cs^+^ resistance growth test on each strain with and without Mg^2+^ addition. Each strain was cultured in Tris medium for 18 h. Dotted line denotes results in the absence of MgCl_2_. Solid line denotes results when 1 mM MgCl_2_ was added. Vertical axis indicates turbidity (OD_600_). Horizontal axis indicates CsCl concentration in the medium. Error bars indicate SD for three independent experiments.

Tris medium (pH 8.0) was supplemented with 100–1,200 mM Cs^+^ plus 1 mM Mg^2+^. A Cs^+^ resistance growth test in the conditions supplemented with 1 mM MgCl_2_ indicated that Mut5 and Mut7 recovered Cs^+^ resistance to the same level as that of the TS-1 wild type (Figure 4). Therefore, it is suggested that Mg^2+^ plays an important role in the Cs^+^ resistance of TS-1. Mut5 and Mut7 might have been Cs^+^-sensitive as they could not take in sufficient Mg^2+^ because of the mutation in each MgtE.

### 3.6. Cs^+^ resistance growth and intracellular Mg^2+^ concentration of *Escherichia coli* Mach1/pGEM-03028

**Figure 5:**
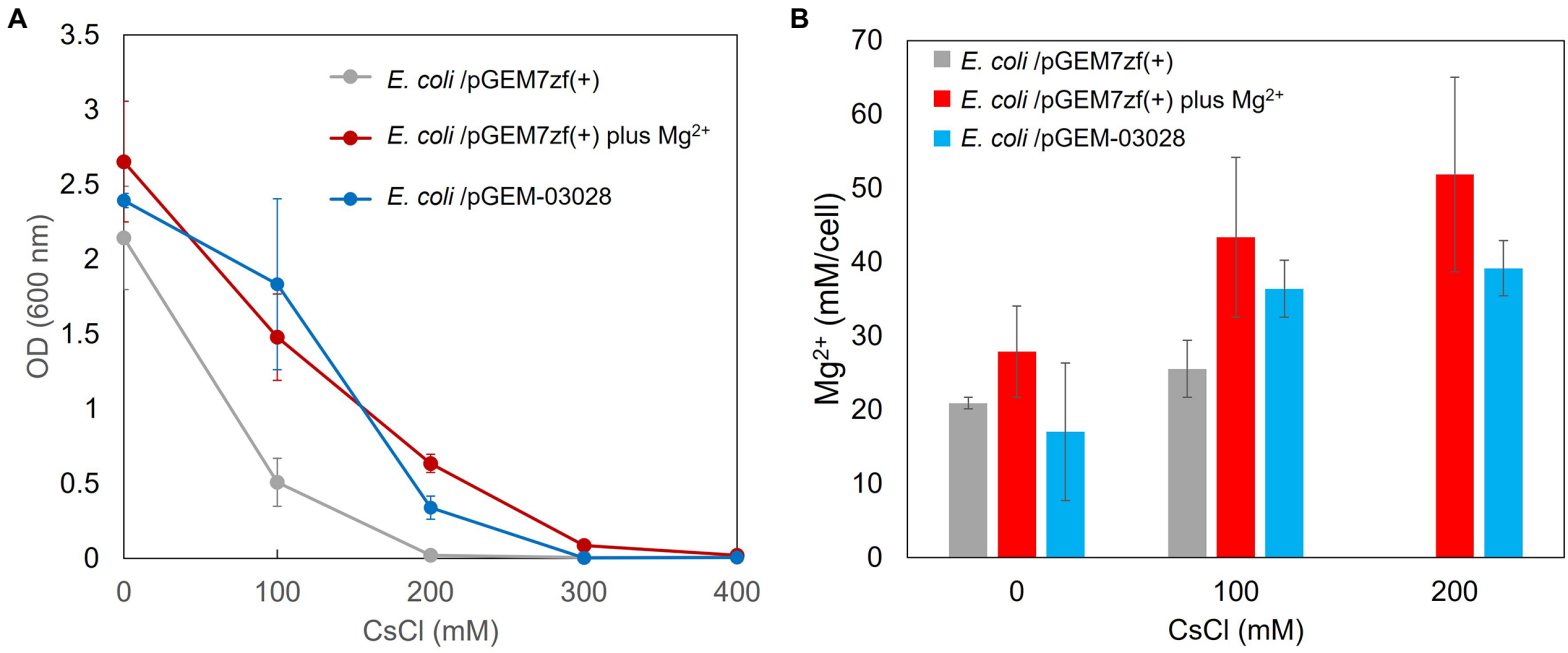
Cs^+^ resistance growth test **(A)** and intracellular Mg^2+^ concentrations **(B)** of *Escherichia coli* Mach1 harboring pGEM-03028 and *E. coli* Mach1 harboring pGEM7zf (+) in presence of Mg^2+^. **(A)** Turbidity (OD_600_) of *E. coli* Mach1/pGEM-03028 in LB medium and Mach1/pGEM7zf (+) in LB medium plus 50 mM MgCl_2_ at each CsCl concentration and after 16 h of culturing. Error bars indicate SD for three independent experiments. *E. coli* Mach1/pGEM7zf (+) was used as the negative control. **(B)** Intracellular Mg^2+^ content at each CsCl concentration for *E. coli*/pGEM-03028 cultured in LB medium (pH 7.5) for 16 h. Vertical axis indicates intracellular Mg^2+^ concentration. Horizontal axis indicates CsCl concentration in the medium. Error bars indicate SD for three independent experiments. An empty vector pGEM7zf (+) was used as the negative control.

The Cs^+^ resistance growth test was conducted, and intracellular Mg^2+^ concentration was measured to determine whether MgtE expression in pGEM7zf (+) improved Mg^2+^ uptake and Cs^+^ resistance. OD_600_ was measured after 16 h of culturing in media containing various CsCl concentrations (Figure 5A). The intracellular Mg^2+^ concentrations are shown in Figure 5B. The minimum inhibitory concentration (MIC) of CsCl was 200 mM for *E. coli* Mach1 harboring the negative control pGEM7zf (+). The *E. coli* Mach1 harboring the MgtE-encoding plasmid pGEM-03028 exhibited resistance to 200 mM CsCl and its MIC was increased to 300 mM. Hence, its Cs^+^ resistance was augmented. The intracellular Mg^2+^ concentration was ~20 mM in the CsCl-free medium both in the presence and absence of MgtE expression. In the latter case, however, the intracellular Mg^2+^ concentration increased by ~50% at elevated CsCl concentrations, whereas in the former case, it increased to ~100%. Thus, *E. coli* Mach1 absorbs Mg^2+^ in the presence of Cs^+^. Furthermore, MgtE expression improved both Mg^2+^ uptake and Cs^+^ resistance.

### 3.7. Cs^+^ resistance in *Escherichia coli* KNabc/pBAD-00475 in the presence of Mg^2+^

A Cs^+^ resistance growth test was conducted to confirm whether MTS1_00475 expression improved Cs^+^ resistance in *E. coli* KNabc. MTS1_00475 is a *cshA* gene product that encodes a low-affinity Cs^+^/H^+^ antiporter. In previous studies, KNabc/pBAD-00475 displayed the same degree of Cs^+^ resistance as that of the negative control KNabc/pBAD24 (vector) (Koretsune et al., 2022). In previous study, the reason why MTS_00475 (CshA) could not improve the Cs^+^ resistance of *E. coli* KNabc is that *E. coli* cannot grow in the presence of 200 mM CsCl, and the apparent *K*_m_ value of CshA for Cs^+^ is 250 mM (pH 8), which is a low affinity. Therefore, it was thought that *E. coli* could not grow at Cs^+^ concentrations at which CshA functions (Koretsune et al., 2022).

**Figure 6:**
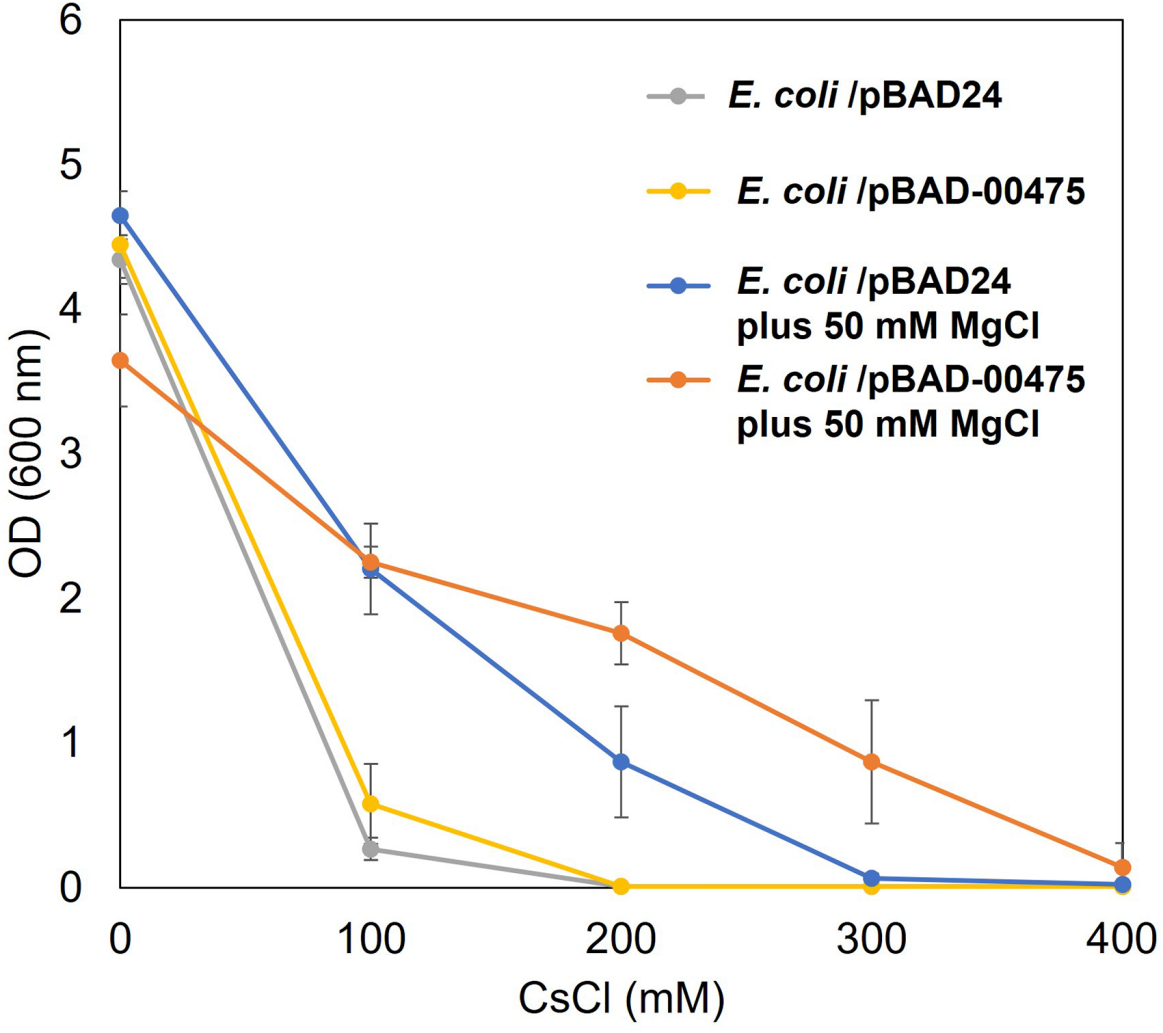
Cs^+^ resistance growth test on *Escherichia coli* KNabc harboring pBAD-00475 in the presence of Mg^2+^. Turbidity (OD_600_) of *E. coli* KNabc/pBAD-00475 in LBK medium and *E. coli* KNabc/pBAD-00475 in LBK medium plus 50 mM MgCl_2_ at each CsCl concentration and after 16 h of culturing. Error bars indicate SD for three independent experiments. *E. coli* KNabc/pBAD24 was used as the negative control.

The addition of Mg^2+^ to the medium improved Cs^+^ resistance in *E. coli* (Figure 5A). Therefore, we conducted a Cs^+^ resistance growth test to evaluate whether the combination of *E. coli* KNabc/pBAD-00475 plus Mg^2+^ addition enhanced Cs^+^ resistance. In the presence of Mg^2+^, *E. coli* KNabc/pBAD-00475 growth was observed at 300 mM CsCl (Figure 6). These observations suggest that the MTS1_00475 product encoding the Cs^+^/H^+^ antiporter CshA effluxed Cs^+^ at this condition and improved its Cs^+^ resistance.

## 4. Discussion

### 4.1. Identification of a novel Cs^+^ resistance-related gene in Cs^+^-sensitive mutants

We attempted to obtain a Cs^+^-sensitive mutant from the TS-1 strain. We used EMS to induce Cs^+^ resistance through chemical mutagenesis. We obtained Mut5 and Mut7 which had reduced Cs^+^ resistance and no *CshA* mutation. We determined that the reversion frequencies in Mut5 and Mut7 were 5.8 × 10^−9^ and 1.9 × 10^−8^, respectively. Unstable and stable mutations had reversion frequencies of ≥1.0 × 10^−6^ and ≤ 1.0 × 10^−8^, respectively. Here, we successfully obtained a Cs^+^-resistant revertant. Analyzes of Cs^+^ resistance in the Cs^+^-sensitive mutant and its revertants disclosed that the phenotype varied with the tested strain.

Mut5 grew at the same rate as that of the wild type in the absence of Cs^+^. The growth of both strains was inhibited by 200 mM Cs^+^. Hence, the Cs^+^ resistance-related gene was mutated in Mut5 and differed from those in Mut3 and Mut4 (Koretsune et al., 2022). A whole-genome analysis revealed no mutation in the MTS1-00475 region of Mut5 or Mut7. A comparison of Mut5 and Mut5R disclosed a back mutation in the *mgtE* region. Mut5R grew at the same rate as that of the wild type whether or not Cs^+^ was present in the medium. As Cs^+^ resistance was fully rescued in Mut5R, *mgtE* is a vital Cs^+^ resistance-related gene.

Mut7 exhibited slower growth than the wild type in the absence of Cs^+^, and its growth was inhibited by 100 mM Cs^+^. Furthermore, its revertant Mut7R grew at the same rate as that of the wild type regardless of Cs^+^ addition. In the absence of Cs^+^, Mut7R rescued both growth and Cs^+^ resistance. A comparison of the mutation sites in Mut7 and Mut7R revealed that the back mutation occurred in the *mgtE* region similar to that in Mut5. Therefore, *mgtE* is critical for growth and Cs^+^ resistance.

### 4.2. Importance of Mg^2+^ in TS-1 Cs^+^ resistance

It was proposed that Mut5 and Mut7 were sensitive to Cs^+^ because of mutations in their MgtE Mg^2+^ uptake system (MTS1_03028). Mg^2+^ addition to the medium increased the intracellular Mg^2+^ concentration and restored Cs^+^ resistance to the wild-type level. Hence, Mut5 and Mut7 were sensitive to Cs^+^ as they could not incorporate sufficient Mg^2+^. For this reason, adequate Mg^2+^ uptake is required for Cs^+^ resistance in TS-1.

The Group 2 metal magnesium is an essential trace element in many living organisms. Magnesium maintains ribosome structure, stabilizes cell membranes and Mg^2+^-dependent enzymatic reactions involved in ribosome synthesis, and has numerous functions in animals, fungi, microorganisms, plants, and other life forms (Akanuma et al., 2014). Depletion of Mg^2+^ in *E. coli* growth medium induces ribosome disassembly, and binding of ribosomal subunits to form 70S ribosomes is forced at Mg^2+^ concentrations above 15 mM. When the Mg^2+^ concentration is lowered below 1 mM, the 70S ribosomes dissociate and eventually unfold. A single *E. coli* ribosome contains at least 170 Mg^2+^. The presence of divalent and monovalent cations stabilizes the tertiary structure of 23S rRNA by mediating interactions between its structural domains (Akanuma et al., 2014). Based on the above, ribosomes are speculated to be one of the candidates affected by intracellular Cs^+^ elevation. Therefore, in the future, we would like to verify the stability of the ribosome structure in the presence of Cs^+^ in the TS-1 and the Cs^+^-sensitive mutant strains using methods such as sucrose density-gradient centrifugation.

The present study demonstrated that 50 mM MgCl_2_ addition improved Cs^+^ resistance in *E. coli*. On the other hand, TS-1 recovered Cs^+^ resistance when one mM MgCl_2_ was added to the Cs^+^-sensitive mutants. Wild-type strain TS-1 is already highly resistant to Cs^+^ due to MgtE and Cs^+^/H^+^ antiporter (CshA). However, the importance of Mg^2+^ in TS-1 was discovered by the MgtE mutant obtained in this study. *E. coli*, which does not have a Cs^+^ efflux mechanism such as a Cs^+^/H^+^ antiporter, is presumed to acquire Cs^+^ resistance by requiring higher concentrations of Mg^2+^. It is speculated that it might have a similar effect in other microbial taxa. The next challenge is to verify whether Mg^2+^ constitutively enhances Cs^+^-resistance in different microorganisms.

To the best of our knowledge, no prior studies have shown that Mg^2+^ plays an essential role in microbial Cs^+^ resistance. However, the present work was the first to reveal this association in TS-1.

### 4.3. Cs^+^ resistance mechanism in TS-1

In our recent study, we reported using intracellular Cs^+^ (CsCl) concentrations of 0 mM to 400 mM in *E. coli* and strain TS-1 (Koretsune et al., 2022). When CsCl was added to the culture medium of *E. coli*, intracellular Cs^+^ concentrations accumulated at levels similar to or higher than extracellular concentrations. Conversely, strain TS-1 suppressed intracellular Cs^+^ concentrations below 150 mM even when exposed to 400 mM CsCl. Comprehensively judging this result and those of the present study, there are two central Cs^+^ resistance mechanisms in strain TS-1. First, strain TS-1 acquired resistance to ≤200 mM Cs^+^ by absorbing Mg^2+^ *via* MgtE. Second, when strain TS-1 was exposed to >200 mM Cs^+^, Cs^+^ was excreted *via* the low affinity Cs^+^/H^+^ antiporter CshA. High-concentration Cs^+^-resistant bacterium strain TS-1 utilizes Mg^2+^ accumulation by Mg^2+^ transporter and Cs^+^ efflux by Cs^+^/H^+^ antiporter to maintain Cs^+^ resistance against low to high Cs^+^ concentrations in the external environment. Several studies have endeavored to isolate Cs^+^-resistant bacteria. To our knowledge, however, this study is the first to elucidate a physiological Cs^+^ resistance mechanism.

## 5. Conclusion

In the present work, we isolated two Cs^+^-sensitive mutants from the *Microbacterium* sp. strain designated TS-1, identified revertant strains, compared their mutation sites, and identified novel Cs^+^ resistance-related gene candidates. The strains Mut5 and Mut7 were obtained in the process of isolating Cs^+^-sensitive strains lacking any mutation in *MTS1_00475*. It was confirmed that Mut5 and Mut7 harbored mutations in *MTS1_03028*. When a single mutation was induced in *MTS1_00475* or *MTS1_03028*, Cs^+^ resistance was significantly reduced. Thus, both of these genes play critical roles in Cs^+^ resistance. Future research should aim to develop a strategy for utilizing TS-1 and other bacteria with the aforementioned Cs^+^ resistance mechanism in the bioremediation of Cs^+^-contaminated soil adjacent to nuclear power plants, radioactive ore tailings, and others.

## Data availability statement

The datasets presented in this study can be found in online repositories. The names of the repository/repositories and accession number(s) can be found at: NCBI - https://www.ncbi.nlm.nih.gov/sra/DRR328007; https://www.ncbi.nlm.nih.gov/sra/?term=DRR328008; https://www.ncbi.nlm.nih.gov/sra/?term=DRR328009; https://www.ncbi.nlm.nih.gov/sra/?term=DRR328010.

## Author contributions

MI designed the research and wrote the manuscript. YI, TK, EI, MT, and MI conducted the research. TK, YI, and MI analyzed the data. All authors contributed to the article and approved the submitted version.

## Funding

This work was supported by a grant for the Toyo University Top Priority Research Promotion Program and the Toyo University intellectual property practical application promotion program.

## Conflict of interest

The authors declare that the research was conducted in the absence of any commercial or financial relationships that could be construed as a potential conflict of interest.

## Publisher’s note

All claims expressed in this article are solely those of the authors and do not necessarily represent those of their affiliated organizations, or those of the publisher, the editors and the reviewers. Any product that may be evaluated in this article, or claim that may be made by its manufacturer, is not guaranteed or endorsed by the publisher.

## Footnotes

^1^ https://sourceforge.net/projects/samtools/files/samtools/0.1.6/

^2^ https://github.com/samtools/bcftools/releases/download/1.16/bcftools-1.16.tar.bz2

^3^ https://ddbj.nig.ac.jp/

^4^ http://www.geneius.de/GENEius/

^5^ https://www.ddbj.nig.ac.jp/index-e.html

^6^ https://blast.ncbi.nlm.nih.gov/Blast.cgi?PAGE=Proteins

^7^ https://www.genome.jp/tools-bin/clustalw

^8^ https://www.genome.jp/tools-bin/ete

^9^ https://services.healthtech.dtu.dk/service.php?TMHMM-2.0

^10^ https://services.healthtech.dtu.dk/service.php?TMHMM-2.0
