# Supplementary material for: A magnesium transporter is involved in the cesium ion resistance of the high-concentration cesium ion-resistant bacterium Microbacterium sp. TS-1
Source: Front Microbiol. 2023 Feb 23;14:1136514. doi: 10.3389/fmicb.2023.1136514 (PMC9995610; doi:10.3389/fmicb.2023.1136514)
Supplement: Supplementary file 1 [file Data_Sheet_1.PDF]

Additional file 1

A magnesium transporter is involved in the cesium ion resistance of the high-concentration cesium ion-resistant bacterium *Microbacterium* sp. TS-1

Yoshiki Ishida[1#], Takahiro Koretsune[1#], Eri Ishiuchi[2], Miyu Teshima[2], and Masahiro Ito[1,2,3,4*]

[1]Graduate School of Life Sciences, Toyo University, Oura-gun, Gunma 374-0193 Japan
[2]Faculty of Life Sciences, Toyo University, Oura-gun, Gunma 374-0193 Japan
[3]Bio-nano electronics Research Center, Toyo University, Kawagoe, Saitama 350-8585 Japan
[4]Bio-resilience research project (BRRP), Toyo University, Oura-gun, Gunma 374-0193 Japan
[#]These authors contributed equally to this work.

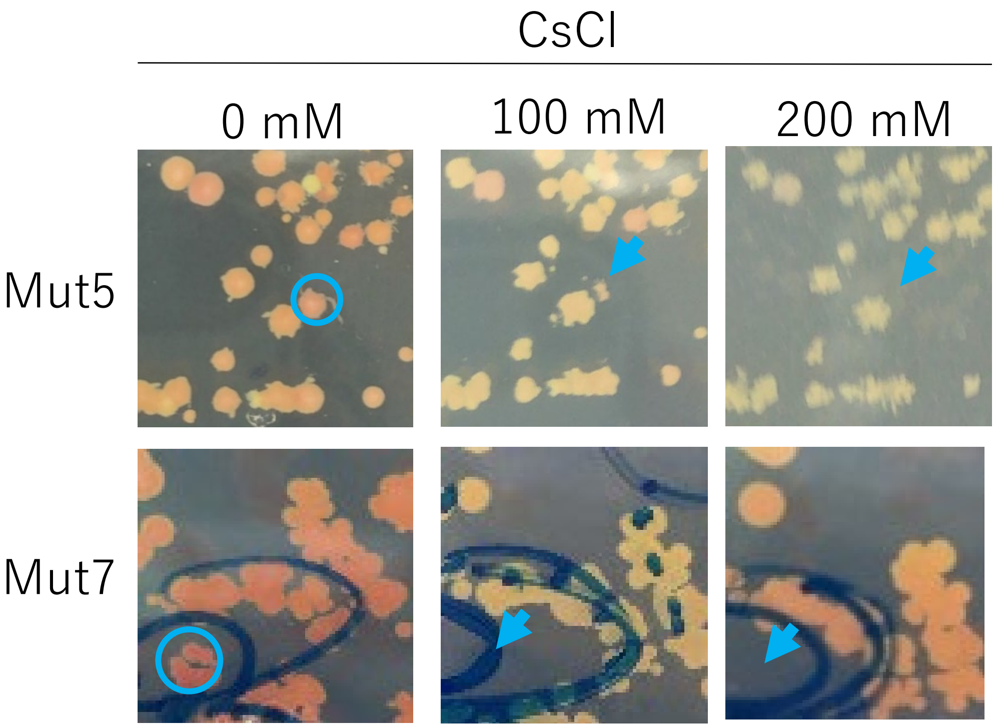

Supplementary Figure S1. Isolation of Cs^+-sensitive mutants via replica plating

Colonies of Cs^+-sensitive mutants obtained using the EMS treatment and replica plating are shown. From the left to the right panel: CsCl-free complex agar medium; complex agar medium containing 100 mM CsCl; complex agar medium containing 200 mM CsCl. pH = 8.0. Blue arrows indicate Cs^+-sensitive colonies.
